# Identification of Supramolecular Structures of Porphyrin Polymer on Single-Walled Carbon Nanotube Surface Using Microscopic Imaging Techniques

**DOI:** 10.3390/polym15061439

**Published:** 2023-03-14

**Authors:** Ahmed I. A. Abd El-Mageed, Takuji Ogawa

**Affiliations:** 1 Chemistry Department, Faculty of Science, GALALA University, Galala City 43711, Egypt; 2 Colloids & Advanced Materials Group, Chemistry Department, Faculty of Science, Minia University, Minia 61519, Egypt; 3 Chemistry Department, Graduate School of Science, Osaka University, Machikaneyama 1-1, Toyonaka 560-0043, Osaka, Japan

**Keywords:** porphyrin, AFM, SWNT, supramolecular structure, polymer, TEM

## Abstract

Although the supramolecular structure of porphyrin polymers on flat surfaces (i.e., mica and HOPG) has been extensively studied, the self-assembly arrays of porphyrin polymers on the SWNT (as curved nanocarbon surfaces) have yet to be fully identified and/or investigated, especially using microscopic imaging techniques, i.e., scanning tunneling microscopy (STM), atomic force microscopy (AFM), and transmission electron microscopy (TEM). This study reports the identification of the supramolecular structure of poly-[5,15-bis-(3,5-isopentoxyphenyl)-10,20-bis ethynylporphyrinato]-zinc (II) on the SWNT surface using mainly AFM and HR-TEM microscopic imaging techniques. After synthesizing around >900 mer of porphyrin polymer (via Glaser-Hay coupling); the as-prepared porphyrin polymer is then non-covalently adsorbed on SWNT surface. Afterward, the resultant porphyrin/SWNT nanocomposite is then anchored with gold nanoparticles (AuNPs), which are used as a marker, via coordination bonding to produce a porphyrin polymer/AuNPs/SWNT hybrid. The polymer, AuNPs, nanocomposite, and/or nanohybrid are characterized using 1H-NMR, mass spectrometry, UV-visible spectroscopy, AFM, as well as HR-TEM measuring techniques. The self-assembly arrays of porphyrin polymers moieties (marked with AuNPs) prefer to form a coplanar well-ordered, regular, repeated array (rather than wrapping) between neighboring molecules along the polymer chain on the tube surface. This will help with further understanding, designing, and fabricating novel supramolecular architectonics of porphyrin/SWNT-based devices.

## 1. Introduction

Porphyrin is derived from the ancient Greek word “Porphura,” which describes the color purple. Porphyrins are a broad group of intensely colored organic dyes that can be found in nature or made synthetically [1,2]. Porphyrins play a crucial role in biochemical processes in vivo as well as metabolism, where they are naturally present in two well-known vital biochemical molecules, i.e., hemoglobin (iron II porphyrin) and chlorophyll (magnesium II porphyrin). Therefore, life would be impossible without porphyrins and their metal derivatives. Consequently, understanding these porphyrin systems can aid our understanding of a variety of essential biological processes, including oxygen binding in hemoglobin, biological catalysis, and the light absorption step in the photosynthesis process.

Porphyrins and their derivatives may be synthesized with various functional groups, including anchoring groups that can be directly linked to metal electrodes. Porphyrins are architecturally adaptable, and there are different chemical methods that may be used to combine various building blocks, including aldehydes, pyrroles, dipyrromethanes, dipyrromethenes, and linear tetrapyrroles, which can be utilized in the synthesis schemes. In addition to being exceedingly stable in solution and at high temperatures, porphyrins may be dissolved in both organic and aqueous solvents [3].

Porphyrins, related compounds, and other polymers become significant candidates in many potential applications (i.e., electronic devices, physics, and many materials science applications) [4,5,6,7,8,9,10,11,12,13,14] as they have remarkable physical and chemical features, in addition, they can be simply synthesized with a wide substituent range.

Likewise, carbon nanotubes (especially single-walled carbon nanotubes (SWNTs)) have attracted much more attention in nanoscience and nanotechnology due to their unusual properties compared to other materials and high applicability [15]. Accordingly, SWNTs can be involved in many applications, i.e., molecular and nano-electronics [16,17,18], sensing [19,20,21,22], medicine [23,24], and as catalysts [25,26].

Based on their size and shape, nanoparticles have distinct physical, chemical, optical, and catalytic properties [27,28]. The integration of SWNT, porphyrins, and nanoparticles will lead to a crucial improvement in the composite characteristics and/or properties [12,29,30,31,32].

Our group has made a lot of efforts to explore the supramolecular structures of porphyrin polymers on flat surfaces (i.e., mica and HOPG) [33,34,35,36], but still the self-assembly arrays of porphyrin polymers on the SWNT (as curved nanocarbon surfaces) have not yet been fully identified and/or investigated (although there have been some attempts from our group and others [37,38,39,40]) especially using microscopic imaging techniques, i.e., scanning tunneling microscopy (STM), atomic force microscopy (AFM), and transmission electron microscopy (TEM).

In our previous study [31] we successfully reported that porphyrin monomer can be self-assembled on the SWNT surface, forming a regular pattern wrapping around the SWNT. Consequently, we could successfully identify (for the first time) the absolute handedness chirality of SWNT via the supramolecular structure of porphyrin monomer using STM imaging technique under ambient conditions.

We have tried to identify the supramolecular structures of porphyrin polymers on SWNT surface using STM imaging technique as we did it using porphyrin monomer [10,31], but unfortunately, we could not as the polymer probably may form a complicated 2D structure which cannot be possibly identified using STM especially under ambient conditions.

Herein, we have succeeded in identifying the supramolecular structure of poly-[5,15-bis-(3,5-isopentoxyphenyl)-10,20-bis-ethynylporphyrinato]-zinc (II) on the SWNT surface using mainly AFM and HR-TEM microscopic imaging techniques. Firstly, around ~ >900 mer porphyrins were non-covalently adsorbed on SWNT surface via π-π stacking as well as van der Waal interactions. Afterward, the resultant porphyrin/SWNT nanocomposite was then anchored with gold nanoparticles (AuNPs), which were used as a marker, via coordination bonding to produce a porphyrin polymer/AuNPs/SWNT hybrid. The self-assembly arrays of porphyrin polymers (marked with AuNPs) were then distinguished mainly using AFM and HR-TEM microscopic imaging techniques, where the porphyrin moieties prefer to form a coplanar well-ordered regular repeated array (rather than wrapping) between neighboring molecules along the polymer chain on the tube surface.

## 2. Experimental Section

### 2.1. Synthesis of the Target Molecules

**Scheme 1:**
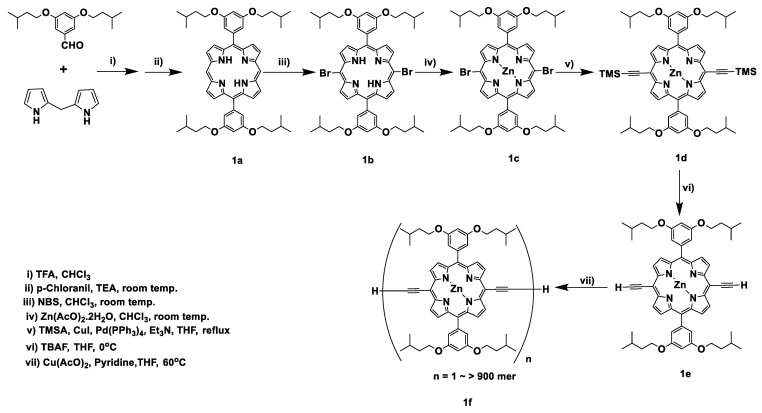
Procedures for synthesizing porphyrin polymer.

The target porphyrin molecules were synthesized using previously reported method [33] (with modification especially in the polymerization step) in which copper catalyzed oxidative coupling was used to produce the final polymer target with molecular wt. ~ >900 mer as shown in Scheme 1. Other materials and reagents were mentioned in detail in the SI.

### 2.2. Polymer Purification

Porphyrin polymer was purified and/or isolated using Shimadzu LC-6A high-performance analytical gel permeation chromatography (GPC) equipped with a diode array detector (MD-2015, JASCO) and two serially connected GPC KF-804L columns. Tetrahydrofuran (THF) was used as an eluent with a flow rate of 1.0 mL/min. All the other characterization techniques are listed in detail in the SI.

### 2.3. Purification of Raw-HiPCO SWNT

Before use, the raw HiPCO-SWNT was purified using our previously reported method [29], in which we removed the amorphous carbon (by heating) as well as the metal catalysts (by refluxing with HCl) from the sample, followed by neutralization of the sample by washing with an aqueous solution of NaHCO_3_. Lastly, the purified SWNT was dried and kept for the next preparation step. The complete purification is displayed in Figure S1.

### 2.4. Fabrication of Porphyrin Polymer/SWNT Nanocomposite

The porphyrin polymer/SWNT nanocomposite was prepared as depicted in Figure S2, by mixing purified SWNT (0.1 mg) with porphyrin polymer solution in THF (≥900 mer), followed by 1-h sonication and another 1-h centrifugation (at 2000 G). After removing the highly bundled SWNT, we have collected around 2 mL of the supernatant. The collected supernatant was then filtered (through a MILLIPORE membrane filter, 0.1 µm mesh), rinsed with 100 mL CHCl_3_ to remove any non-adsorbed porphyrins, dried, and kept for further use.

### 2.5. Synthesis of t-Dodecanethiol-Pyridine Ethanethiol Capped AuNPs

We have synthesized gold nanoparticles as *t*-dodecanethiol-pyridine ethanethiol-protected gold nanoparticles (Scheme S1), as previously reported by our group [36], using the modified Brust method [41]. In this method, an aqueous solution of HAuCl_4_ (186 mg/15 mL H_2_O) is mixed with a toluene solution of tetraoctylammonium bromide solution (1000 mg in 40 mL toluene) with continuous stirring. The resultant mixture is composed of two layers, the toluene layer with a red orange color (after receiving the AuCl_4_¯ ion), and the colorless aqueous layer. After removing the aqueous layer, 85 mg of *t*-dodecanethiol was added to the solution, followed by a color change from red orange to colorless. To the resultant solution, an aqueous solution of NaBH_4_ (212 mg/12.5 mL H_2_O) was added dropwise with stirring at ~30 °C for 3 h where the solution color turns into wine red. The resultant mixture was then concentrated, centrifuged in ethanol, dissolved the ppt in toluene, reprecipitated again with ethanol, followed by centrifugation to produce *t*-dodecanethiol AuNPs (~23 mg). The resultant solution of *t*-dodecanethiol AuNPs in toluene (10 mg in 10 mL) was then mixed with 4-pyridineethanethiol (10 mg, 0.72 µmol) with stirring for 24 h. The product was then centrifuged, followed by dissolving the residue in methanol, and centrifuged again. By removing the residue and evaporating the solvent, the final solid was washed many times with toluene, dried as a black powder (~ 8 mg).

### 2.6. Fabrication of Porphyrin Polymer/AuNPs/SWNT Nanohybrid

The porphyrin polymer/AuNPs/SWNT nanohybrid was prepared by sonicating the polymer/SWNT nanocomposite (~ 0.1 mg) with methanolic solution of AuNPs (0.1 mg in 1 mL) for 30 min, followed by centrifugation, filtration, washing with MeOH (100 mL), dried under N_2_ gas, and finally kept for the microscopic measurements (as presented in Figure S3).

## 3. Results and Discussion

### 3.1. Synthesis of the Target Molecules

**Figure 1:**
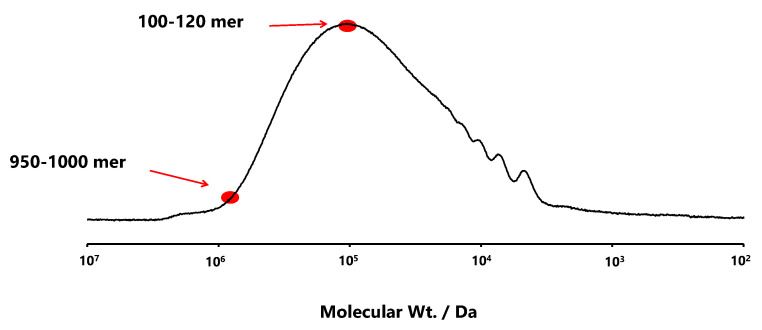
Analytical GPC chart of porphyrin polymer in THF, at room temperature, using a standard polystyrene calibration curve. The arrows refer to the formation of ~ 100–120 and 950–1000 mer at around 10^5^ and 10^6^ Da, respectively.

The target porphyrin molecules (**1a**–**e**) were synthesized using our reported method [33]. The characterizations (i.e., ^1^HNMR, mass, and UV spectroscopies) of the synthesized molecules (**1a**–**e**) are fully presented in the SI (Figures S4–S8). However, the porphyrin polymer molecule (**1f**) was synthesized with a slight modification in the polymerization step using Glaser-Hay coupling (i.e., copper catalyzed oxidative coupling) of diethynylporphyrin (**1e**) with Cu(OAc)_2_ in pyridine under overnight heating at 60 °C, as displayed in Scheme 1. The resultant product was then quenched with water giving precipitate, which was then collected after filtration and washing with methanol. The polymer species were isolated by high-performance analytical gel permeation chromatography (GPC) in THF (as a mobile phase) using a standard polystyrene calibration curve. The molecular weight of the final porphyrin polymer was determined at around 10^6^ Da, which corresponds to the formation of ~ 950–1000 mer, as obviously depicted in Figure 1, based on the molecular weight of porphyrin monomer (918.5 Da).

**Figure 2:**
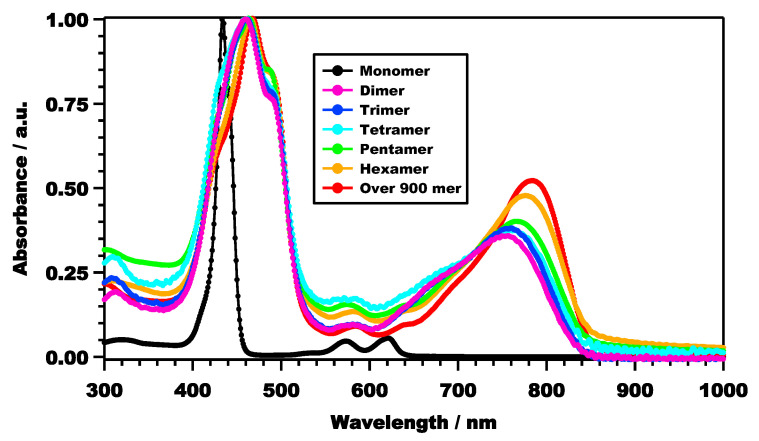
UV-visible spectra of porphyrin monomer (**1e**), dimer, trimer, tetramer, pentamer, hexamer, and porphyrin polymer (**1f**), measured in THF. The spectra show an obvious chemical red-shift in both the Soret band and Q-band compared with the monomer (**1e**) due to the polymerization as well as a high degree of conjugation of the separated products of polymer species.

Figure 2 displays the UV-visible spectra of porphyrin monomer (**1e**), dimer, trimer, tetramer, pentamer, hexamer, and porphyrin polymer (**1f**), measured in THF. The spectra show an obvious chemical red-shift in the Soret band (around 80 nm) as well as Q-band (~ 200 nm) compared with the monomer (**1e**), due to the polymerization as well as a high degree of conjugation of the separated products of polymer species, which agrees with the results reported elsewhere [33].

### 3.2. Supramolecular Structure of Porphyrin Polymer on HOPG Surface Characterized by AFM

To identify the supramolecular structures of porphyrin polymer on SWNT (as curved nanocarbon) surface, a closer look at the porphyrin polymer supramolecular structures on HOPG (as flat nanocarbon) surface is important. Consequently, AFM measurements were performed for the porphyrin polymer on the HOPG surface. The sample was prepared using two different techniques, i.e., simple drop-casting and two-bottle soaking methods. In the simple drop-casting technique, the polymer solution (i.e., 20 μL) was dissolved in THF:H_2_O (10:1) and then drop-casted onto the HOPG surface, followed by spontaneous evaporation of the solvent (as illustrated in Figure S9a). However, in the two-bottle soaking method, the HOPG was soaked in the first bottle containing polymer solution (dissolved in THF:H_2_O (10:1)), followed by immersing the first bottle in another bottle filled with H_2_O for 30 min under heating at 60 °C (Figure S9b), followed by spontaneous evaporation of the solvent.

**Figure 3:**
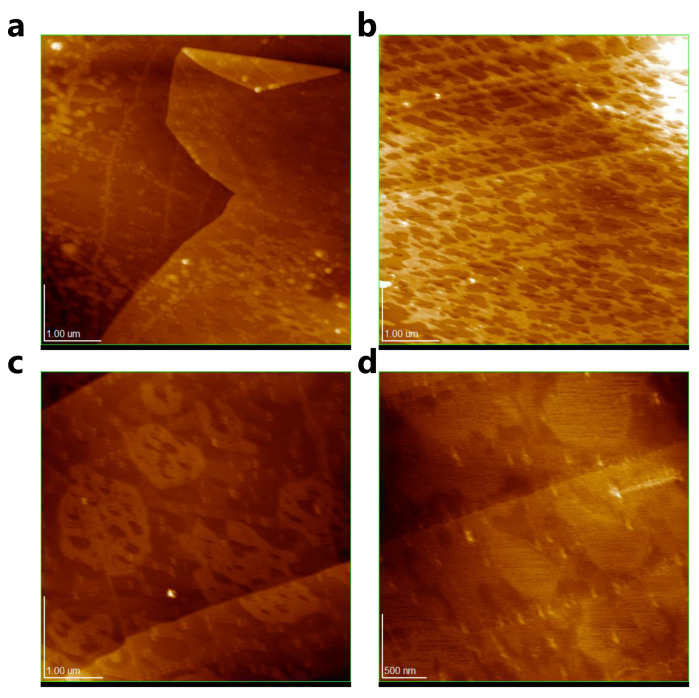
(**a**–**d**) Typical AFM images of porphyrin polymer (over-900 mer) on the HOPG surface collected using tapping mode under ambient conditions.

Figure 3a–d displays the AFM images of porphyrin polymers (over-900 mer) on HOPG surface collected using tapping mode under ambient conditions. The porphyrin polymer in Figure 3a,b (prepared using the two-bottle soaking method) shows a lump-like structure; however, in Figure 3c,d (prepared using the simple drop-casting method), the polymer displays a linear-like structure, with a height of ~ 1 nm, as displayed in Figure S10a,b. In our previous study [33], we have successfully investigated the self-assemble structure of the same porphyrin polymer (with lower molecular weight i.e., over-200 mer) on HOPG surface using AFM imaging technique, in which the porphyrin polymer was self-assembled in the form of wire-like structure. However, in this study, we could not observe the same wire-like structure, probably due to the higher molecular weight of the porphyrin polymer (i.e., over-900 mer) that led to more aggregation (as in Figure 3a,b) and/or a linear-like structure (as in Figure 3c,d).

### 3.3. Supramolecular Structure of Porphyrin Polymer on SWNT Surface

**Figure 4:**
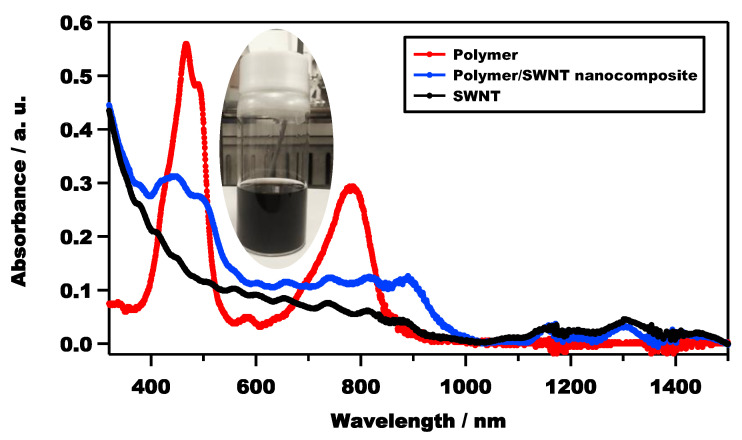
UV-visible spectra of porphyrin polymer (**1f**), SWNT, and polymer/SWNT nanocomposite measured in THF. The spectra show an obvious chemical shift in both the Soret band and Q-band compared with the polymer (**1f**) due to non-covalent interaction. The inset displays the polymer/SWNT nanocomposite sample dispersed in THF.

The raw HiPCO-SWNTs were purified (by removing amorphous carbon as well as metal catalysts) using our previously reported method [29,30]. The porphyrin polymer/SWNT nanocomposite was fabricated by sonicating porphyrin polymer with purified SWNT in THF, followed by centrifugation, filtration, and drying (Figure S2). Figure 4 presents the UV-visible spectra of porphyrin polymer (**1f**), SWNT, and polymer/SWNT nanocomposite measured in THF. The polymer/SWNT nanocomposite spectra include the characteristic peaks of both porphyrin polymer and SWNT, as an indication for the successful functionalization of SWNT with the polymer molecules.

Additionally, the polymer/SWNT nanocomposite spectra show a clear chemical shift in both the Soret band and Q-band compared with the polymer (**1f**) due to the non-covalent interactions (i.e., π-π stacking and/or van der Walls) between SWNT and polymer molecules. The chemical shift could also occur from an extended conjugation caused by the enhanced coplanarity of the porphyrin polymer units bound to the rigid sidewall structure of the nanotube. The polymer/SWNT nanocomposite sample dispersed in THF is displayed in the inset of Figure 4.

**Figure 5:**
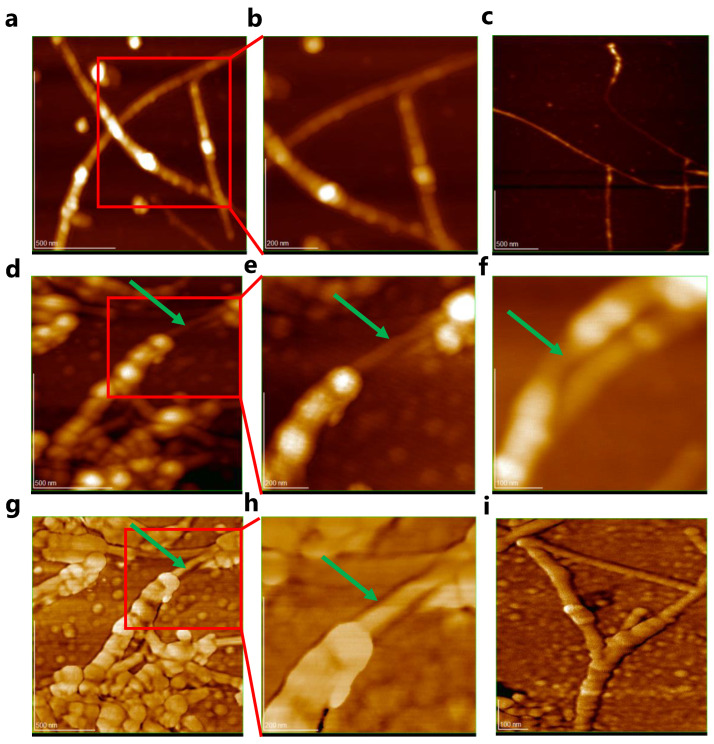
Typical AFM images (**a**–**f**) (topographic) (**g**–**i**) (phase) of polymer/SWNT nanocomposite on mica surface collected under ambient conditions. The green arrows in the images (**d**–**h**) refer to bare SWNT.

Figure 5 depicts the typical AFM images (a–f) (topographic) (g–i) (phase) of polymer/SWNT nanocomposite on mica surface collected under ambient conditions. The images demonstrate an obvious SWNT debundling effect owing to the adsorption of porphyrin polymer on the SWNT surface, which is an indication for the successful functionalization of SWNT with the polymer molecules, which agrees with the UV-visible measurements (Figure 4). Nevertheless, there are some SWNT parts that are not totally functionalized with the polymer molecules, as marked by the green arrows in the images (d–h), which refer to bare SWNT.

However, in some images (i.e., a, b, d, e, g, and i), the polymer molecules are aligned in a regular pattern on the tube surface. For more confirmation, we have tried to identify the supramolecular structures of porphyrin polymers on SWNT surface using STM imaging technique as we did it using porphyrin monomer [10,31], but unfortunately, we could not as the polymer probably may form a complicated 2D structure which cannot be possibly identified using STM especially under ambient conditions. Therefore, it was worthy to use gold nanoparticles (AuNPs) as markers after anchoring with porphyrin molecules via coordination bonding to produce a porphyrin polymer/AuNPs/SWNT hybrid that can be possibly identified using HR-TEM as well as AFM with clearer images, which will be discussed in detail in the following section.

### 3.4. Supramolecular Structure of Porphyrin Polymer Marked with AuNPs on SWNT Surface

Firstly, we synthesized the protected gold nanoparticles (AuNPs) in the form of *t*-dodecanethiol-pyridineethanethiol-AuNPs by using the ligand-exchange method [36] (modified from the original method [41]), in which an exchange was conducted between *t*-dodecanethiolAuNPs and 4-pyridineethanethiol in toluene, as illustrated in Scheme S1. The synthesized AuNPs were characterized by UV-visible spectroscopy as well as HR-TEM. The UV-visible (Figure S11a) shows the characteristic plasmon peak of AuNP at 521 nm, which agrees with the reported results [42]. However, the average diameter of the synthesized AuNPs determined from the TEM measurement (Figure S11b) is about 2.6 ± 0.2 nm, which agrees with our reported results [36].

The porphyrin polymer/AuNPs/SWNT nanohybrid was then prepared by sonicating the polymer/SWNT nanocomposite with a methanolic solution of AuNPs for half an hour, followed by centrifugation, filtration, washed with MeOH (100 mL), dried under N_2_ gas, and finally kept for the microscopic measurements (as presented in Figure S3). The porphyrin/SWNT nanocomposite was anchored with gold nanoparticles (AuNPs), which were used as markers, via coordination bonding to produce the porphyrin polymer/AuNPs/SWNT nanohybrid.

**Figure 6:**
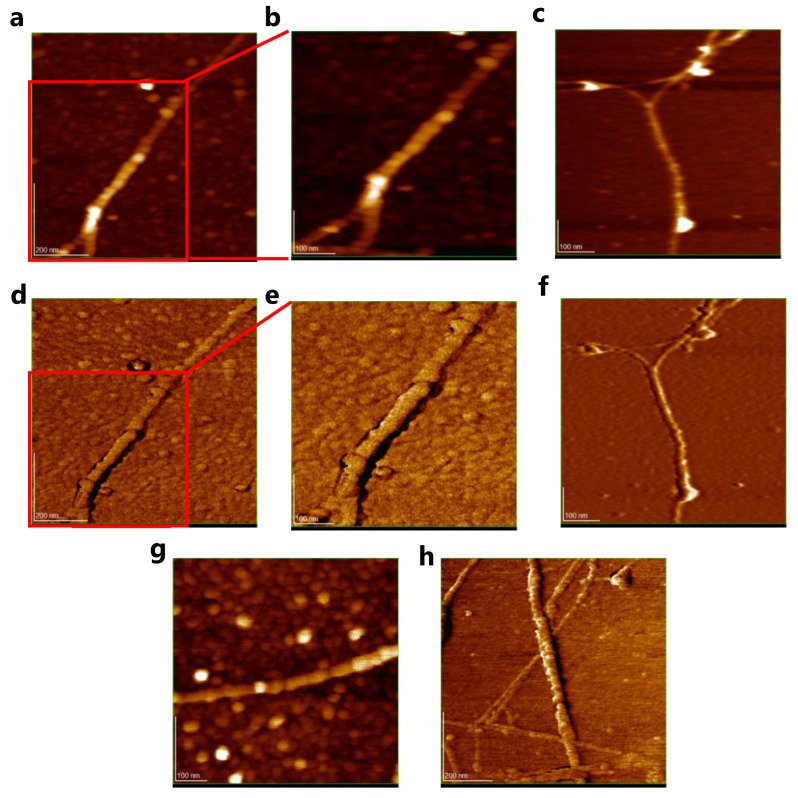
Typical AFM images (**a**,**b**,**c**,**g**) (topographic) and (**d**,**e**,**f**,**h**) (phase) of porphyrin polymer/AuNPs/SWNT nanohybrid on mica surface collected under ambient conditions.

Figure 6 displays the typical AFM images (a, b, c, g) (topographic) and (d, e, f, h) (phase) of porphyrin polymer/AuNPs/SWNT nanohybrid on mica surface collected under ambient conditions. The images demonstrate a clear SWNT debundling effect, which agrees with the UV-visible measurements (Figure 4) as well as AFM measurements of polymer/SWNT nanocomposite (Figure 5). Using AuNPs as a marker is a convenient way to construct the ordered molecular assemblies on the SWNT surface, where we can clearly see the AuNPs are aligned along the SWNTs. Moreover, the images also display obvious self-assembly arrays of porphyrin polymers (marked with AuNPs) on the tube surface.

**Figure 7:**
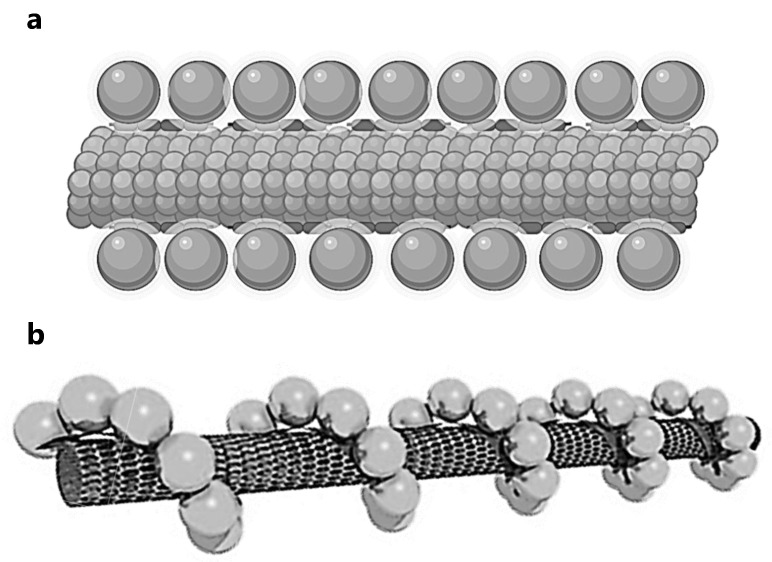
Proposed models for the possible alignment of porphyrin polymer molecules (marked with AuNPs) on SWNT surface: (**a**) porphyrin molecules (marked with AuNPs) may form a coplanar, well-ordered, regular, repeated array, and (**b**) porphyrin molecules (marked with AuNPs) may wrap along the tube surface, forming a helical structure.

Although porphyrin polymer molecules form a well-ordered regular pattern, it is not fully clear (from AFM images) to judge whether it is a coplanar well-ordered regular repeated array between neighboring molecules (Figure 7a) or the polymer molecules are wrapping along the tube surface, forming a helical structure (Figure 7b). As mentioned previously, we have tried to identify the supramolecular structures of porphyrin polymers on SWNT surfaces using STM imaging techniques, but unfortunately, we could not. Therefore, we decided to use HR-TEM measurements instead.

**Figure 8:**
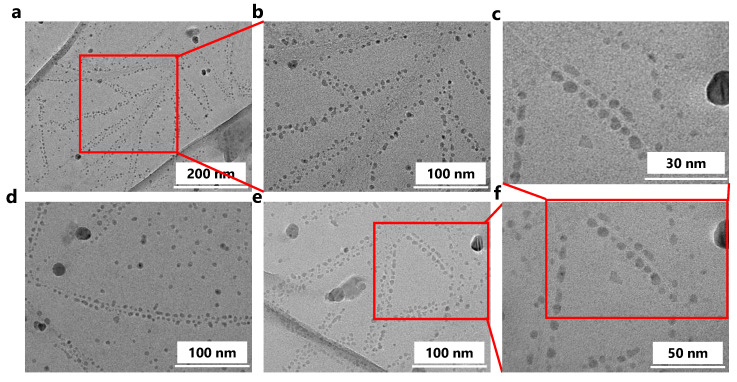
(**a**–**f**) Typical HR-TEM images (with different resolution) of porphyrin polymer molecules (marked with AuNPs) on SWNT surfaces, in which the polymer molecules align in the form of a coplanar well-ordered regular repeated array rather than wrapping along the tube surface.

Figure 8 depicts the typical HR-TEM images of porphyrin polymer molecules (marked with AuNPs) on a SWNT surface. It is obviously shown from the images that the porphyrin polymer molecules self-assemble in the form of a coplanar, well-ordered, regular, repeated arrays (Figure 7a) rather than wrapping along the tube surface. The self-assembly process restricts the porphyrin rotation about the butadiyne links, which increases the coplanarity between neighboring porphyrins along the polymer chain. Additionally, the nanotube acts as a template that restricts torsional disorder within the polymer backbone and induces a coplanar arrangement of porphyrin repeated units, which totally agrees with the previous assumption [40]. It was previously assumed that the polymer molecules could align in the form of a coplanar, well-ordered, regular, repeated array on the tube surface, but unfortunately, with no clear experimental evidence [40], in this study, we succeeded in presenting some experimental proofs for that finding, i.e., HR-TEM as well as AFM measurements.

## 4. Conclusions

In this study, by using mainly AFM and HR-TEM microscopic imaging techniques, we have succeeded in identifying the supramolecular structures of porphyrin polymer, i.e., poly-[5,15-bis-(3,5-isopentoxyphenyl)-10,20-bis-ethynylporphyrinato]-zinc (II) on a single-walled carbon nanotube surface. At first, the SWNT was functionalized with the prepared porphyrin polymer (~ >900 mer) through π-π stacking and/or van der Waal interactions. Subsequently, the resultant porphyrin/SWNT nanocomposite was then anchored with gold nanoparticles (AuNPs), which were used as a marker, via coordination bonding to produce a porphyrin polymer/AuNPs/SWNT hybrid. The self-assembly arrays of porphyrin polymers (marked with AuNPs) were then distinguished mainly using AFM and HR-TEM microscopic imaging techniques, where the measurements indicated that the porphyrin polymer molecules self-assembled in the form of a coplanar, well-ordered, regular, repeated arrays rather than wrapping along the tube surface. The self-assembly process restricts the porphyrin rotation about the butadiyne links, which increases the coplanarity between neighboring porphyrins along the polymer chain. Additionally, the nanotube acts as a template that restricts torsional disorder within the polymer backbone and induces a coplanar arrangement of porphyrin repeated units. This substantial finding will serve as a helpful key point for further understanding and building new supramolecular architectures on curved nanocarbon surfaces, as well as for designing and fabricating novel molecular architectonics of porphyrin/SWNT-based devices.

## 5. Highlights

- The supramolecular structure of poly-[5,15-bis-(3,5-isopentoxyphenyl)-10,20-bis ethynylporphyrinato]-zinc (II) on the SWNT surface was identified using mainly AFM and HR-TEM microscopic imaging techniques;
- Around >900 mer of porphyrin polymer was synthesized using Glaser-Hay coupling (i.e., copper catalyzed oxidative coupling) of diethynylporphyrin with Cu(OAc)_2_ in pyridine under overnight heating at 60 °C;
- The as-prepared porphyrin was then non-covalently adsorbed on SWNT surface via π-π stacking as well as van der Waal interactions;
- Afterward, the resultant porphyrin/SWNT nanocomposite was then anchored with gold nanoparticles (AuNPs), which were used as a marker, via coordination bonding to produce a porphyrin polymer/AuNPs/SWNT hybrid;
- The self-assembly arrays of porphyrin polymers (marked with AuNPs) moieties prefer to form a coplanar, well-ordered, regular, repeated array (rather than wrapping) between neighboring molecules along the polymer chain on the tube surface;
- This will help with further understanding, designing, and fabricating novel supramolecular architectonics of porphyrin/SWNT-based devices.

## Data Availability

All data generated or analyzed during this study are included in this published article [and its Supplementary Information Files].

## Supplementary Materials

The following supporting information can be downloaded at: https://www.mdpi.com/article/10.3390/polym15061439/s1; Figure S1. SWNT purification; Figure S2. Procedures for preparing porphyrin polymer/SWNT nanocomposite; Scheme S1. Synthesis of t-dodecanethiol-pyridine ethanethiol protected gold nanoparticles; Figure S3. Procedures for preparing porphyrin polymer/AuNPs/SWNT nanohybrid; Figure S4. Characterization of porphyrin molecule 1a. (a) 1H-NMR spectrum in CDCl3, (b) Mass Spectrum, (c) UV-visible spectrum in CHCl3; Figure S5. Characterization of porphyrin molecule 1b. (a) 1H-NMR spectrum in CDCl3, (b) Mass Spectrum, (c) UV-visible spectrum in CHCl3; Figure S6. Characterization of porphyrin molecule 1c. (a) 1H-NMR spectrum in CDCl3, (b) Mass Spectrum, (c) UV-visible spectrum in CHCl3; Figure S7. Characterization of porphyrin molecule 1d. (a) 1H-NMR spectrum in CDCl3, (b) Mass Spectrum, (c) UV-visible spectrum in CHCl3; Figure S8. Characterization of porphyrin molecule 1e. (a) 1H-NMR spectrum in CDCl3, (b) Mass Spectrum, (c) UV-visible spectrum in CHCl3; Figure S9. Sample preparation of porphyrin polymer for AFM measurements using (a) simple drop-casting technique, (b) two-bottle soaking method; Figure S10. Histograms show the height of porphyrin polymer (over-900 mer) assembled on HOPG surface; Figure S11. Characterization of t-dodecanethiol-pyridineethanethiol-AuNPs. (a) UV-visible spectrum in methanol, (b) TEM image.
