# Supplementary material for: Identification of Supramolecular Structures of Porphyrin Polymer on Single-Walled Carbon Nanotube Surface Using Microscopic Imaging Techniques
Source: Polymers (Basel). 2023 Mar 14;15(6):1439. doi: 10.3390/polym15061439 (PMC10051620; doi:10.3390/polym15061439)
Supplement: Supplementary file 1 [file polymers-15-01439-s001.zip › polymers-2119536-supplementary.pdf]

Supplementary Information

Article

Identification of Supramolecular Structures of Porphyrin Polymer on Single-Walled Carbon Nanotube Surface Using Microscopic Imaging Techniques

Ahmed I. A. Abd El-Mageed [1,2,*], and Takuji Ogawa [3]

[1] Chemistry Department, Faculty of Science, GALALA University, Galala City 43711, Suez, Egypt
[2] Colloids & Advanced Materials Group, Chemistry Department, Faculty of Science, Minia University, Minia 61519, Egypt
[3] Chemistry Department, Graduate School of Science, Osaka University, Machikaneyama 1-1, Toyonaka, Osaka 560-0043, Japan; ogawa@chem.sci.osaka-u.ac.jp
* Correspondence: ahmed.abdelmageed@gu.edu.eg

Experimental

Materials and Reagents

All reactions were performed in anhydrous solvents under nitrogen atmosphere using well-dried glasswares in an oven at 90 °C before using. All the solvents were dried and distilled using molecular sieves 4 Å. Column chromatography was performed using silica-gel (spherical, neutral, 63-200 µm, Kishida Chemicals Co., Ltd.). Mica sheets and/or HOPG (Spi-1 Grade 7 × 7 × 1 mm) were purchased from Alliance Biosystems, Inc. Other chemicals and solvents were of reagent grade and used without any further purification. The HiPCO-SWNT sample was purchased from Carbon Nanotechnologies Inc. Patch #: P1-00 (diameter 0.8-1.2 nm and length around 100-1000 nm). The target porphyrin molecules were synthesized as shown in Scheme 1.

Characterization Techniques

Mass spectra were recorded using a Shimadzu AXIMA-CFR MALDI-TOF mass spectrometer. UV/Visible adsorption spectra were carried out on a Shimadzu UV-3150 double-beam spectrophotometer. ^{1}H-NMR was performed using an NMR spectrometer (500 MHz, JEOL, Japan). As an internal standard, tetramethylsilane (TMS) was used to adjust the NMR signals. Atomic force microscopic measuments were recorded using JEOL SPM-5200 scanning probe microscope (SPM). All the AFM images were taken using tapping mode under ambient conditions on HOPG and/or mica surface. The high-resolution transmission electron microscopy (HR-TEM) images were taken using JEOL JEM-2100 (LaB6-TEM) with applicable range of acceleration voltage: 80–200 kV and maximum resolution of about 200 pm.

Purification of raw-HiPCO SWNT

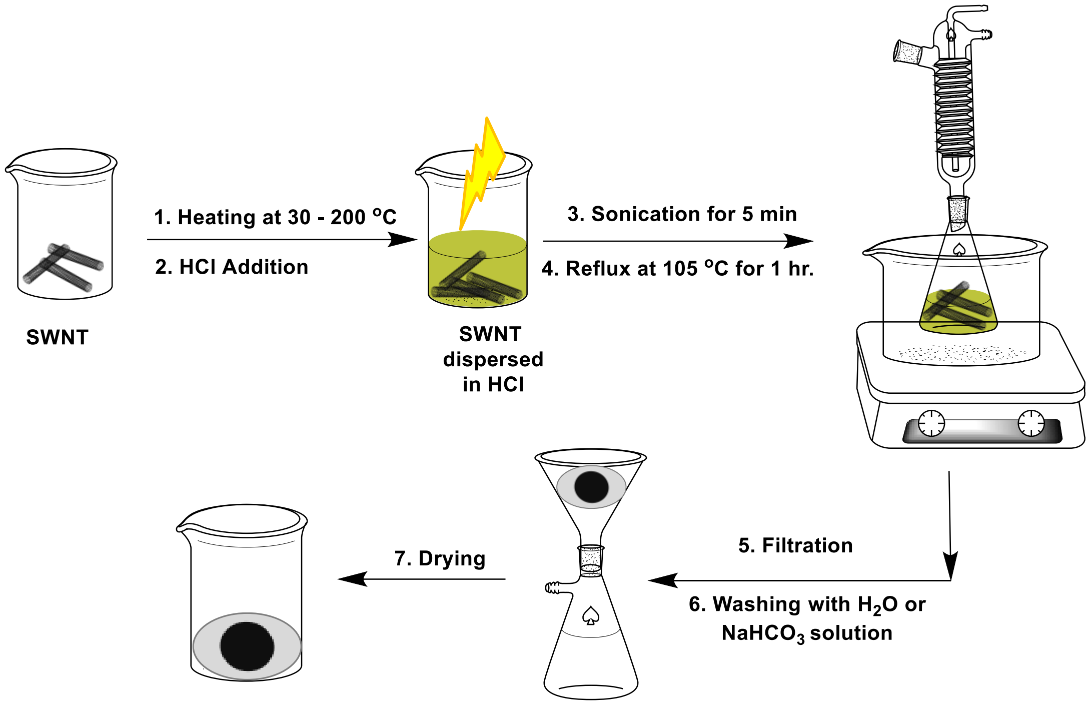

Figure S1. SWNT purification.

Fabrication of porphyrin polymer/SWNT nanocomposite

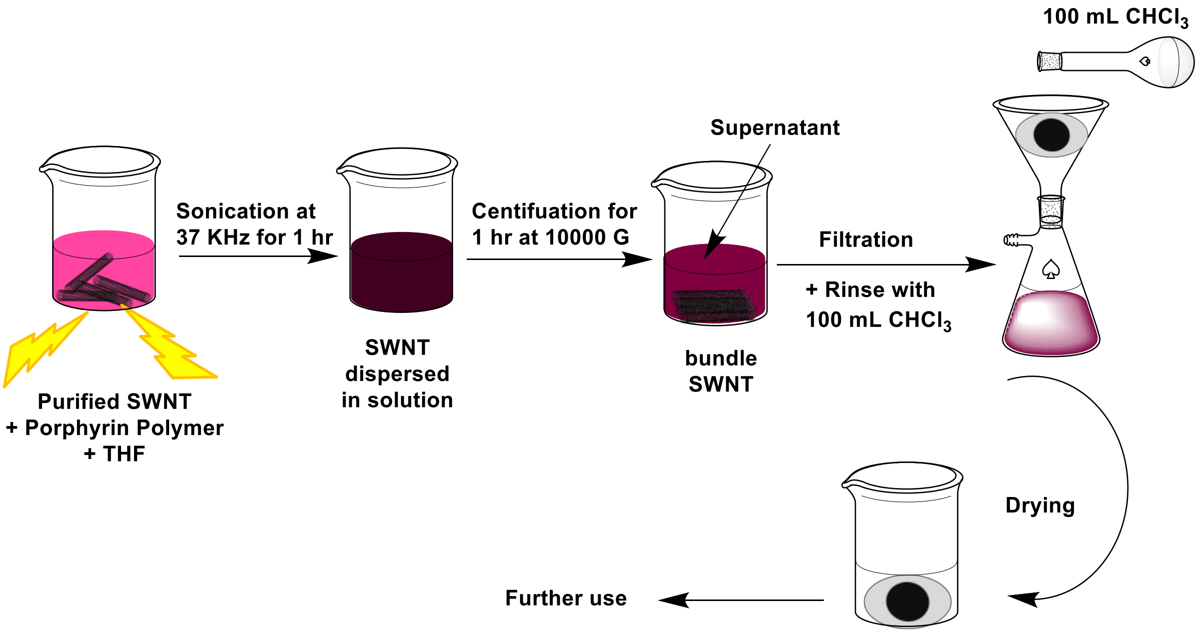

Figure S2. Procedures for preparing porphyrin polymer/SWNT nanocomposite.

Synthesis of *t*-dodecanethiol-pyridine ethanethiol capped AuNPs

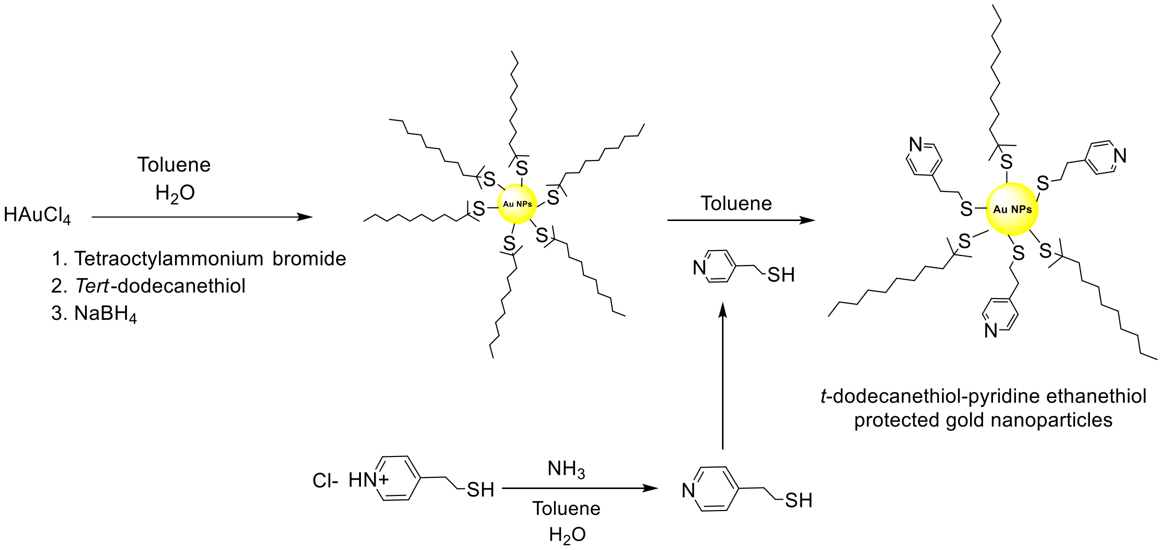

Scheme S1. Synthesis of *t*-dodecanethiol-pyridine ethanethiol protected gold nanoparticles.

Fabrication of porphyrin polymer/AuNPs/SWNT nanohybrid

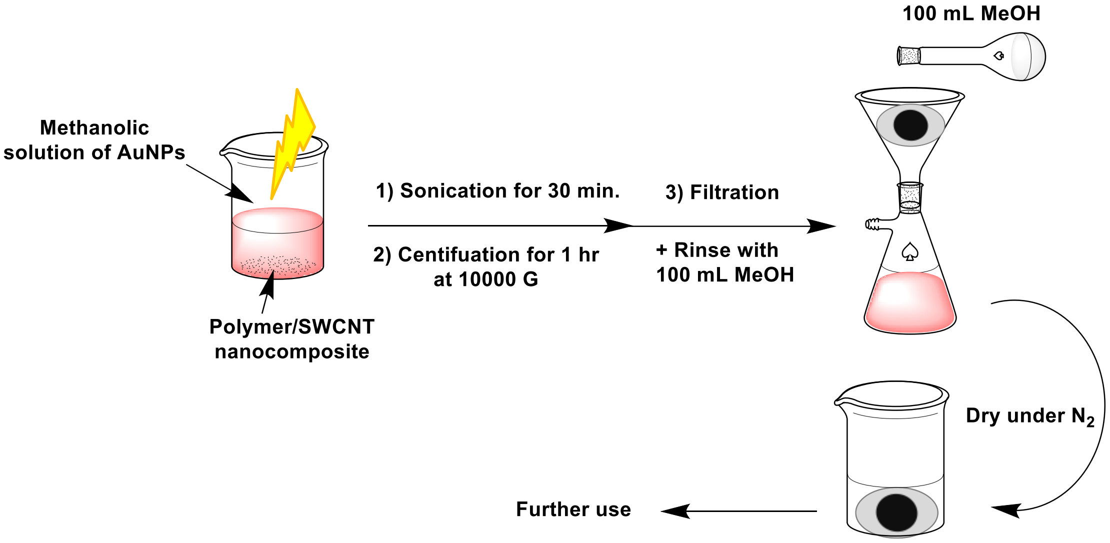

Figure S3. Procedures for preparing porphyrin polymer/AuNPs/SWNT nanohybrid.

Characterizations of porphyrin molecules (1a-e)

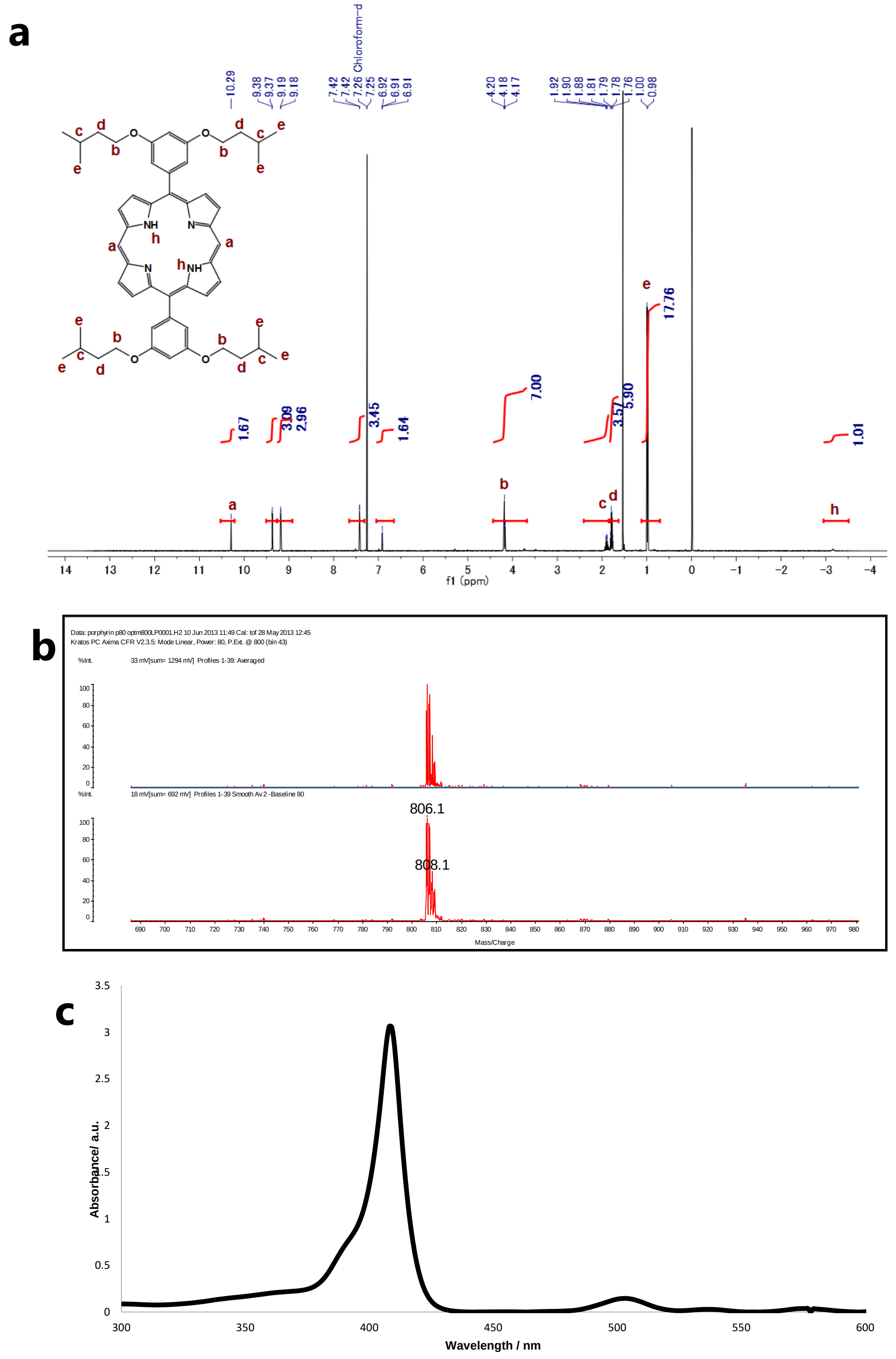

Figure S4. Characterization of porphyrin molecule **1a**. **a)** ^{1}H-NMR spectrum in $CDCl_3$, **b)** Mass Spectrum, **c)** UV-visible spectrum in $CHCl_3$.

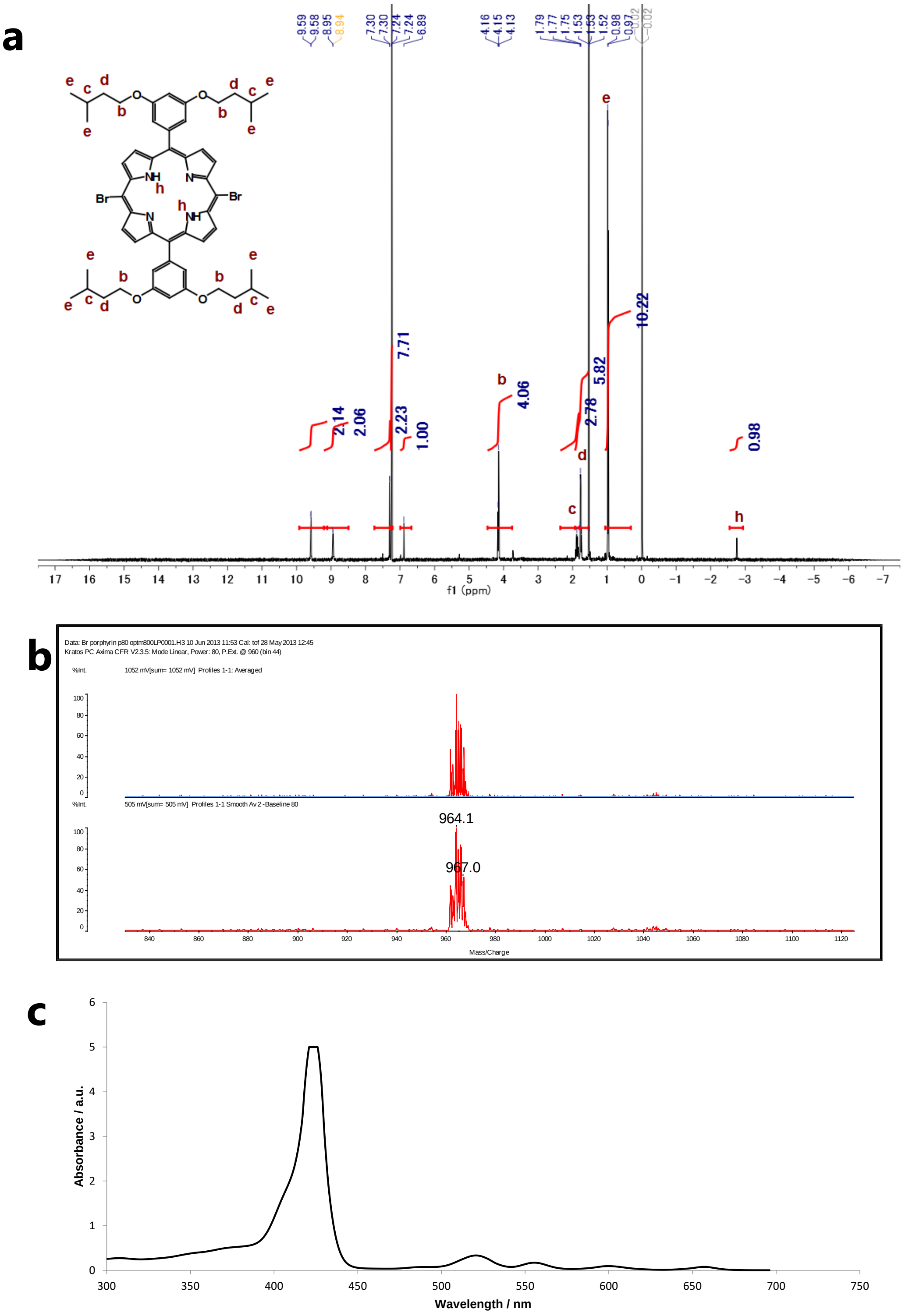

Figure S5. Characterization of porphyrin molecule **1b**. **a)** ^{1}H-NMR spectrum in $CDCl_3$, **b)** Mass Spectrum, **c)** UV-visible spectrum in $CHCl_3$.

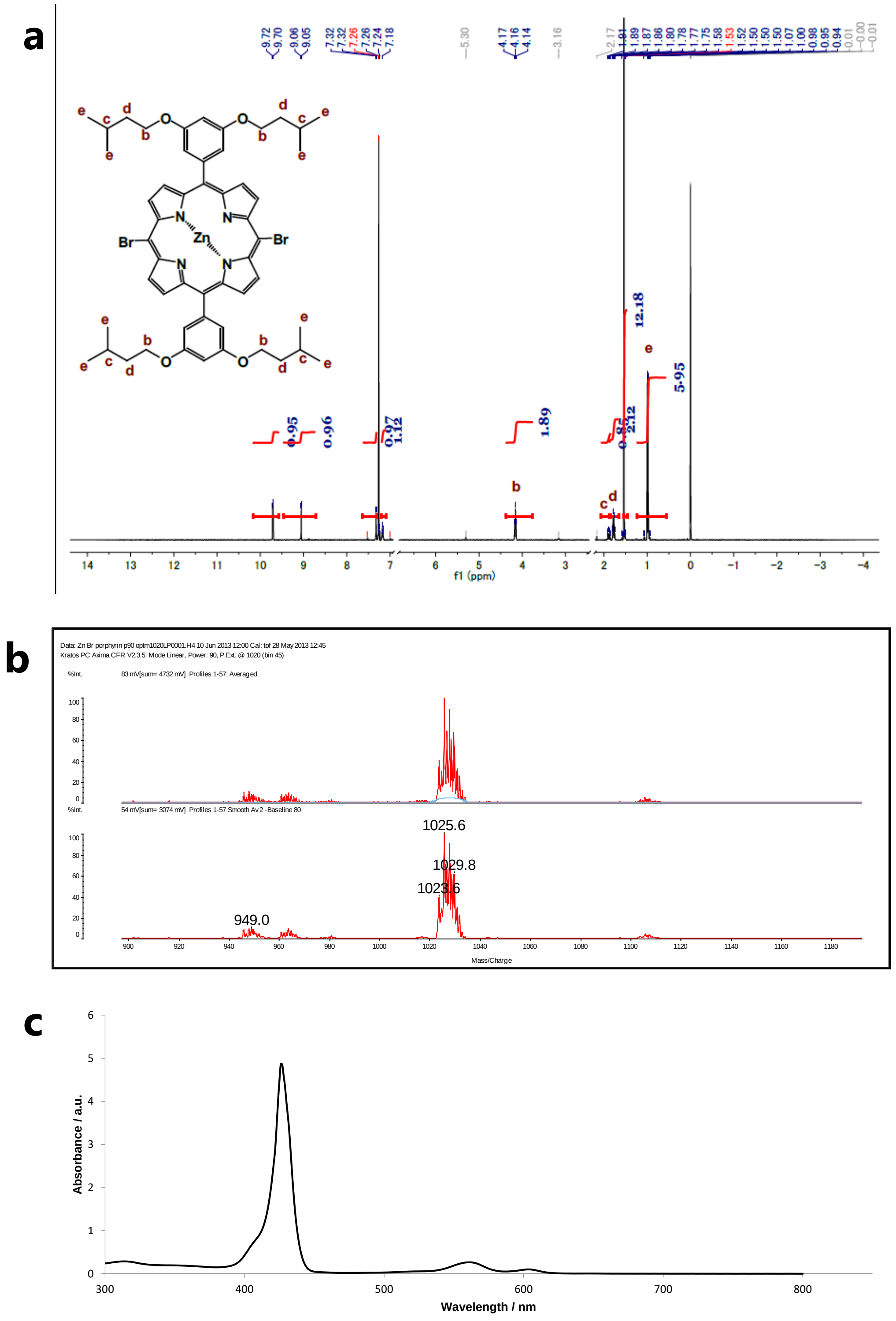

Figure S6. Characterization of porphyrin molecule **1c**. **a)** ^{1}H-NMR spectrum in $CDCl_3$, **b)** Mass Spectrum, **c)** UV-visible spectrum in $CHCl_3$.

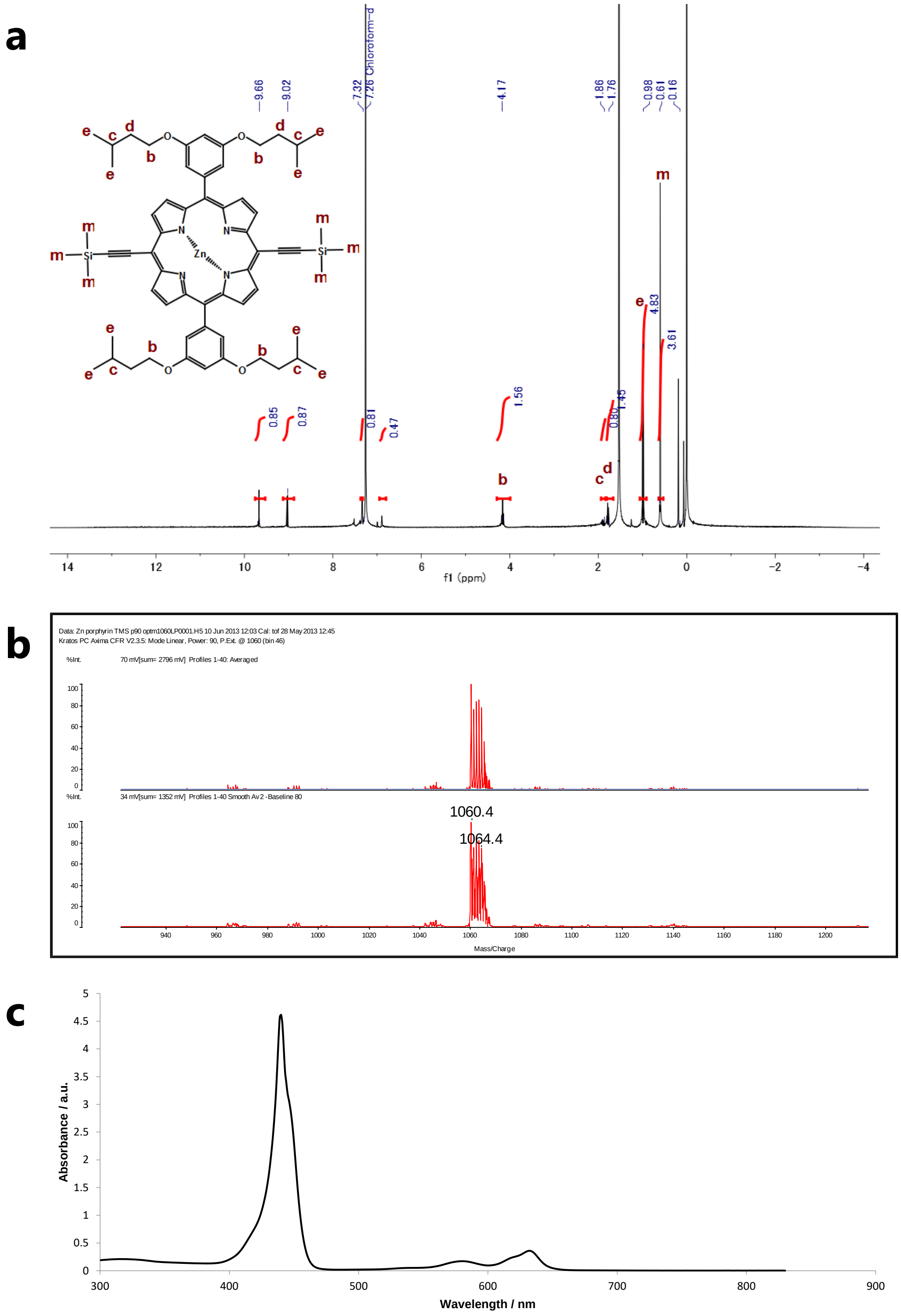

Figure S7. Characterization of porphyrin molecule **1d**. **a)** ^{1}H-NMR spectrum in $CDCl_3$, **b)** Mass Spectrum, **c)** UV-visible spectrum in $CHCl_3$.

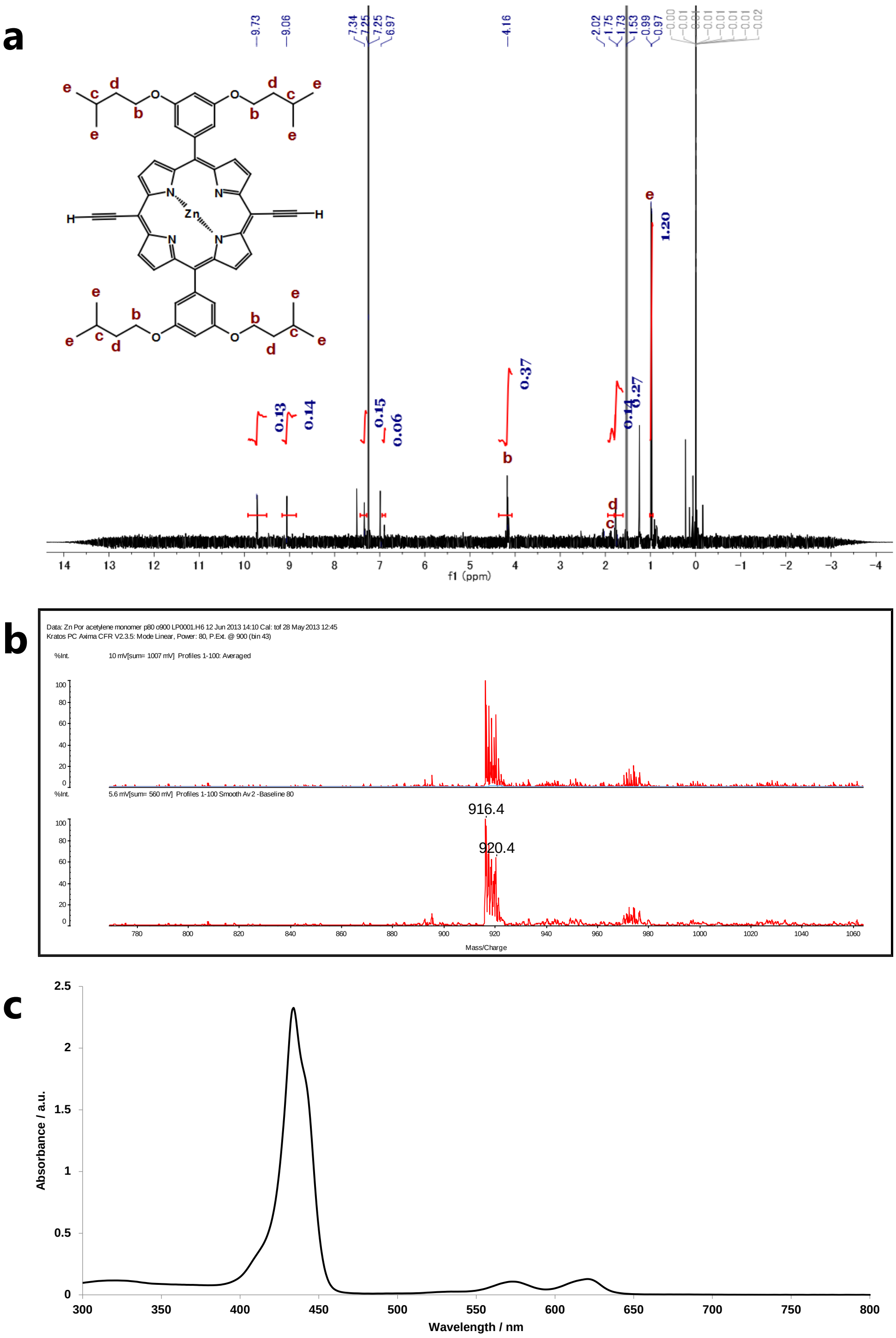

Figure S8. Characterization of porphyrin molecule **1e**. **a)** ^{1}H-NMR spectrum in $CDCl_3$, **b)** Mass Spectrum, **c)** UV-visible spectrum in $CHCl_3$.

Supramolecular structure of porphyrin polymer on HOPG surface characterized by AFM

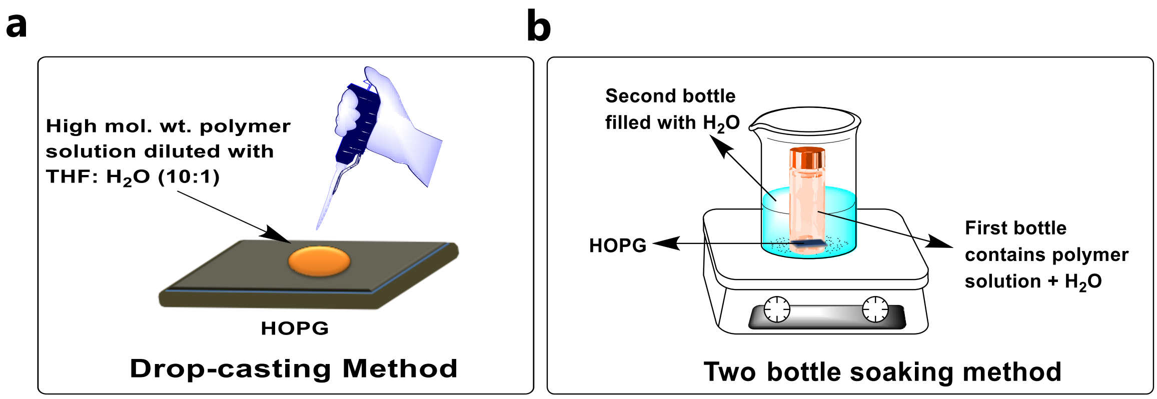

Figure S9. Sample preparation of porphyrin polymer for AFM measurements using **a)** simple drop-casting technique, **b)** two-bottle soaking method.

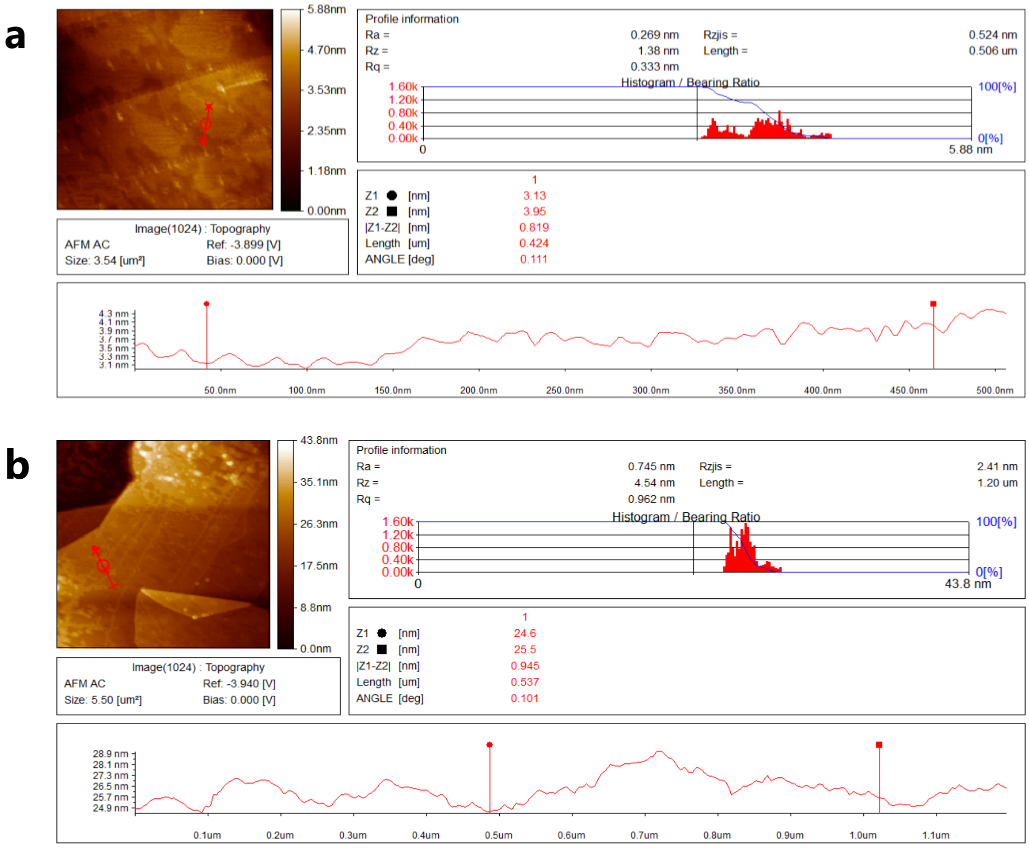

Figure S10. Histograms show the height of porphyrin polymer (over-900 mer) assembled on HOPG surface.

Characterization of *t*-dodecanethiol-pyridineethanethiol-AuNPs

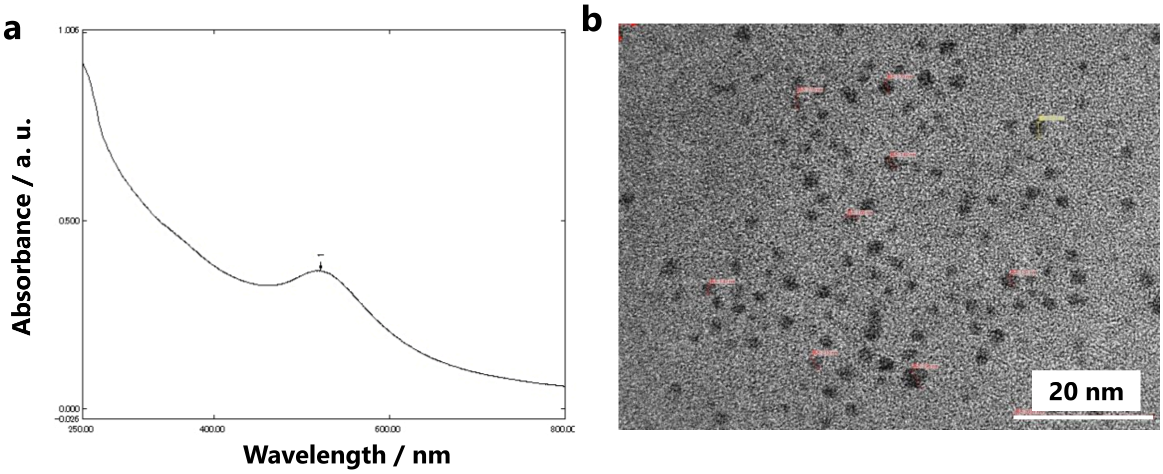

Figure S11. Characterization of *t*-dodecanethiol-pyridineethanethiol-AuNPs. **a)** UV-visible spectrum in methanol, **b)** TEM image.
